# Arbuscular Mycorrhiza in Highly Fertilized Maize Cultures Alleviates Short-Term Drought Effects but Does Not Improve Fodder Yield and Quality

**DOI:** 10.3389/fpls.2019.00496

**Published:** 2019-04-17

**Authors:** Władysław Polcyn, Ewelina Paluch-Lubawa, Teresa Lehmann, Robert Mikuła

**Affiliations:** ^1^Department of Plant Physiology, Faculty of Horticulture and Landscape Architecture, Adam Mickiewicz University, Poznań, Poland; ^2^Department of Animal Nutrition, Faculty of Veterinary Medicine and Animal Science, Poznań University of Life Sciences, Poznań, Poland

**Keywords:** arbuscular mycorrhiza, drought tolerance, fertilization limits, *Zea mays*, *Rhizophagus irregularis*, senescence reversal

## Abstract

Under fertilization levels specific to intensive farming, the impact of compensation of soil nutritional value by arbuscular mycorrhiza (AM) might be limited. Therefore, the question arises whether modern crop varieties, selected for high NPK assimilation rate, are able to gain symbiotic benefits under other challenging field conditions, such as drought. Accordingly, in this study we aimed to evaluate the contribution of *Rhizophagus irregularis* to the drought response of a stay-green corn hybrid in pot cultures equally fertilized until silking, compared to non-mycorrhizal (NM) counterparts. The highest tested fertilization regime not detrimental to the long-term vitality of intraradical hyphae reached the levels recommended for field cultivation of silage corn, except phosphorus application restricted to 60%. Under normal watering, mycorrhiza increased leaf nitrogen and phosphorus acquisition but only in cultures supplied with low NPK levels. At high fertilization levels, only the older leaves retained AM dependency, whereas for other leaf positions the AM-NM differences were leveled out. The similar size and nutritional status of highly fertilized AM and NM cultures, used in this study, eliminated fungal benefits before and during the 2-week drought progression. Nevertheless, mycorrhizal contribution became evident at the time of renewed watering, when AM plants showed much faster reversal of drought-induced leaf senescence symptoms: impaired photosynthesis and nitrogen management. Our results suggest that mycorrhiza can alter drought-induced senescence even in stay-green mutants. Moreover, this effect was apparently not mediated by AM-improved growth but triggered by activation of fungal transport at the time of recovery. Interestingly, the fungal protective potential was shown to be preserved at the expense of lowering AM vesicle number. It can be interpreted as engagement of hyphal nutritional resources targeted to maintain the symbiotic relationship despite the reduced vitality of the host. Finally, we compared the productivity of AM and NM cultures subjected to short-term drought at silking time and further fertilized with moderate or high NPK doses until the grain-filling stage. The yield and nutritive value of green forage showed that alleviation of drought-induced senescence by AM was not sufficient to have a significant positive effect on the final productivity compared to NM plants.

## Introduction

Current crop production is still only a fraction of the yield potential, both in developing and developed countries, due to declining soil fertility and warmer temperatures as well as the drought stress, which is a major environmental constraint in agriculture (St. Clair and Lynch, 2010). Arbuscular mycorrhiza (AM) can affect crop plants productivity in various ways. Traditionally, the improvement is attributed to fungal access to the alternative system of acquisition and transport of water and minerals (P, N, and other nutrients), as the plant root system typically has a limited access to them (Smith and Read, 2008; Treseder, 2013; Hodge and Storer, 2015; Lanfranco et al., 2018). Another widely recognized benefit of fungal symbiosis is an enhancement of plant drought tolerance through water supply, changes to soil hydraulic properties, improved mineral nutrition, root architecture development, altered stomatal conductance and leaf C assimilation, enhanced osmoregulation capacity or oxidative damage protection (Boomsma and Vyn, 2008; Augé et al., 2015; Bitterlich et al., 2018b).

*Rhizophagus irregularis* chosen for our study belongs to generalist symbionts that can colonize a wide variety of host plants and also to species that show an opportunistic behavior in soils exposed to abiotic stress, including drought. The factors contributing to soil abundance of this fungus are high root colonization rate, fast generation of high numbers of spores and yet not recognized interactions with other AM fungi and soil microbes (Lenoir et al., 2016). Species of features similar to *R. irregularis* are suitable as components for large-scale inoculum production programs to introduce AM fungi as biofertilizer for farming technology including maize monocropping (Dias et al., 2018).

Stay-green varieties, frequent among maize hybrids, have the most pronounced ability to avoid yield loss under limiting environmental conditions. The genetic traits of these mutants are responsible for delayed initiation of senescence program during the grain filling stage (Hörtensteiner, 2009). Moreover, the delayed-senescence phenotype is observed to be associated with a higher drought resistance (Gregersen et al., 2013). Therefore, the question arises whether and how the features of stay-green crops may interact with mycorrhizal water and nutrient pathways. Considering AM impact on maize drought tolerance it should be noted that studies including post-flowering period are relatively few in number (Boomsma and Vyn, 2008). Moreover, most of available conclusions were derived from the works of Subramanian et al. (1995) conducted in the second half of the 90’s, on open-pollinated tropical maize cultivar which was not a hybrid *per se*. These experiments were done in a greenhouse setting on drought-tolerant genotype, obtained through recurrent selection as compared to original drought-susceptible cultivar. Both variants, when mycorrhized, responded to drought treatment with higher leaf water potential and stomatal conductance values and recovered quicker from water stress than non-mycorrhizal (NM) counterparts. According to numerous reports (reviewed in Augé, 2001; Augé et al., 2015), such a behavior is in common to AM plants of other species, typically exhibiting greater photosynthetic rates under drought conditions.

Mycorrhizal hyphae could build up the plant drought response potential by a slow additive enhancement of plant nutritional status, improving host growth and development. This would result in mycorrhiza-increased crop productivity under drought. Such improved plant nutrition is the often suggested explanation of drought tolerance in mycorrhizal plants (Rapparini and Peñuelas, 2014). Nevertheless, the fungi forming AM can alter plant–water relations, at least on some occasions, in a way unrelated to growth promotion and long-term nutrient acquisition (Augé, 2001; Augé et al., 2008). Such a mycorrhizal impact could be observed under soil drought stress, when fungal hyphae continue to share with plant host their own water flow. In addition, under higher drought intensities, mycorrhiza lowers soil resistance to water flow, since extraradical hyphae proliferate air-filled pores in areas outside the root zone, bridging spaces where water flow still occurs (Bitterlich et al., 2018a,c). This could result in temporarily enhanced dynamics of water uptake and root hydraulic conductivity, preventing the worsening of leaf water status. This, in turn, could alter gas exchange parameters during drought development and recovery and improve overall plant host water stress tolerance (Augé, 2001; Augé et al., 2015).

In general, AM symbioses increase plant growth in soils of low fertility, whereas mycorrhizal nutritional benefits might be limited under fertilization levels specific to intensive farming (Smith and Smith, 2011). Therefore it is postulated that evaluation of symbiotic benefits should be assessed without sacrificing the yield performance already achievable without fungal support, i.e., not only on the basis of mycorrhizal compensation of poor soil nutritional value. This would help to distinguish plant performance specific to the variation shared by AM and NM plants (i.e., traits working without fungal colonization) from the variation specific to AM (van de Wiel et al., 2016).

Accordingly, in this study we aimed to evaluate the contribution of *Rhizophagus irregularis* to the drought response of a stay-green corn hybrid in pot cultures fertilized until silking with high NPK doses but also intending to limit differences in nutritional status of AM and NM counterparts. To facilitate access to nutrients and prevent from uncontrolled fertilizer concentration changes we designed a soil-free semi-hydroponic system with a mixture of coconut fiber and sand, and frequent irrigation with a water-soluble fertilizer. The substrate mix has excellent water and air exchange properties, allowing to obtain severe but quickly reversible soil drought effects.

In three consecutive pot experiments, we sought to resolve the following issues:

(1) to define fertilization limits and optima for long-term intraradical hyphal vitality and symbiotic coexistence with corn (silage stay-green hybrid ‘Opoka’) until the early generative stage;
(2) to see if mycorrhized plants, adjusted to the nutritional status of NM counterparts, would show alleviated leaf senescence after exposure to progressively increasing drought;
(3) to evaluate if highly fertilized mycorrhizal cultures would present altered fodder quality after drought applied at the particularly sensitive time of silking.

## Materials and Methods

### Plant Culture Conditions

Sterile germinated seeds of *Zea mays* (hybrid Opoka, HR Smolice, Poland) were planted in substrate trays with 25 ml of commercial sterilized peat substrate for vegetables. Two week-old seedlings were transferred into 4 L pots (one plant per pot) filled with coconut fiber (Ceres International)/0.8–1.2 mm sand mixture (3:1, w/w). The experiments were carried out in phytotron under 16 h/8 h light and dark regime and respective temperatures of 25 and 21°C, photosynthetic photon flux density of 900 μE m^-2^s^-1^, and average air humidity of 55%.

Pots were supplemented with tap water to compensate for daily cumulative evapotranspiration (ca. 3 × 200 ml per week at the 8-leaf stage). In this water volume a fully soluble commercial fertilizer of lowered phosphorus content was applied (Kristalon Blue label, Yara Poland) (%): 19 N, 6 P_2_O_5_, 20 K_2_O, 3 MgO, 3 S and addition of microelements (%): 0.025 B; 0.07 Fe (DTPA); 0.04 Mn (EDTA); 0.025 Zn (EDTA); 0.004 Mo; 0.01 Cu (EDTA). The maximal fertilization level (denoted as 1xD), defined as not detrimental for long-term hyphae vitality, was 114 mg N/36 mg P_2_O_5_/120 mg K_2_O/18 mg MgO as expressed in per plant weekly doses. For doses of 0.5xD and 1xD the plants reached 180 cm height and 240 g weight, with shoot cross section of 2 cm, 8 fully green leaves and flowering phase in the typical time for the variety.

The maximal fertilizer volume was reduced to 25% for the first 6 weeks (until the 6-leaf stage) in order to establish fungal colonization of roots. After this period the plants were provided for 4 weeks (until tasseling, BBCH 59 stage, 10 weeks after seeding) with one of four dilutions of 1xD dose **(task 1)**. The visible symptoms of leaf N or P deficiency was specific mainly to 0.125xD fertilization variant and first 4 weeks cultivation on 0.25xD fertilizer dilution. For drought experiments **(task 2)** the cultivation from 6th to 12th week was continued on 1xD fertilization level until silking (63 BBCH stage when pollination begins and ear silks begin to emerge). Soil drought was imposed by withholding watering for several days, followed by renewed fertilizer irrigation. For fodder quality analyses **(task 3)**, the pots were treated from 6th week with half (0.5xD) or maximal (1xD) fertilization volume until half-milkline stage of grain development recommended for silage harvest (85 BBCH stage, approximately 20th week). For each of the fertilization, symbiotic or drought treatments 4–6 plants, each one from separate pot, were used for analyses. The number of treatments and replicates was provided in legends of appropriate figures.

### Mycorrhizal Colonization

Each seedling was inoculated at the time of transfer with suspension of 250 spores of *Rhizophagus irregularis*. The inoculum (Centre for Mycorrhizal Research, The Energy and Resources Institute, New Delhi, India), free of microbiological contaminants, was obtained from monoxenic root organ cultures (Adholeya et al., 2005). For fodder quality analyses the plants were cultivated on the soil taken from preceding AM or NM cultures (i.e., keeping the same microbiological composition, except mycorrhizal component), diluted 1:15 (w/w) with sterile coconut-sand substrate.

The following parameters were assessed in root samples: frequency of fungal structures in the root system (F%), intensity of the mycorrhizal colonization (M%) and arbuscular abundance in colonized parts of root fragments (a%) – the hyphae vitality index. The percentage of mycorrhizal root infection was estimated by visual inspection of fungal colonization after clearing 1cm long root fragments in 10% KOH (24 h, RT) and staining with 0.05% trypan blue in lactic acid (v/v, 24 h, RT). Mycorrhizal parameters (F%, M%, a%) were calculated according to Trouvelot’s method, counted from 150 root fragments^1^ (Trouvelot et al., 1986). Vesicles abundance was quantified as an average number per cm of total root length, counted from 300 to 400 root fragments.

### Leaf and Root Nitrogen and Phosphorus Content

Leaf samples were taken from three vertical canopy positions (counted from top): upper leaves (2nd and 3rd), middle leaves (ear leaf and leaf above the ear) and lower leaves (6th and 7th). For each of these positions the tissue material was collected avoiding the midrib. The data represent averaged values (weight N or P units per leaf dry mass, g 100 g^-1^), taken as mixed samples (*n* = 4), from two adjacent leaves (joined leaf numbers: L2+3, L4+5, and L6+7), using four plants for each fertilization and symbiotic variant. For each technical repetition, 0.5 – 1 g of leaves or roots was dried for 4 h at 103°C to a constant weight in accordance with the applicable AOAC standards, weighed to obtain the dry weight (DW), and then ground into a fine powder. A portion of plant material was used to determine total N concentration according to Kjeldahl method using Kjeltec 8400 Auto Sampler System. The phosphorus content was analyzed after additional 10 h mineralization at 500°C, followed with hydrogen peroxide, nitric acid and ultrasonic treatment, according to ammonium molybdate colorimetric method (PN-ISO 6491) in conjugation with UV-visible Nicolet Evolution 300 spectrophotometer.

### Fluorimetric Estimation of Leaf Nitrogen Management Efficiency

Leaf nitrogen status was estimated with chlorophyll/flavonoids ratio (NBI, Nitrogen Balance Index) using Dualex 4 Scientific (Force-A, Orsay, France) fluorimeter. The chlorophyll level was estimated from red light (710 nm) transmittance, automatically corrected for the interference from other leaf structures by division by transmittance at the reference wavelength of 850 nm (Cerovic et al., 2012). The amount of flavonoids is estimated from difference in chlorophyll fluorescence induced by UV (375 nm) and red light (650 nm) since only UV is affected by the presence of flavonoids. The readings covered upper surface of apical half of two leaves from middle nodes (ear leaf and leaf above) and data was presented as the averaged values of 50–80 sampling points from four plants for each symbiotic and drought or fertilization variant.

### Chlorophyll Fluorescence Induction Kinetics

The fluorescence induction kinetics of chlorophyll a were estimated using a pulse amplitude modulated (PAM) fluorimeter (FMS1, Hansatech) according to Lichtenthaler et al. (2005). Pre-darkened leaf reveals minimal fluorescence (F_0_) under weak far-red (735 nm) light pulses (for 5 s). Measurement of other fluorescence parameters are based on transient fluorescence induction kinetics known as the Kautsky effect (Fracheboud and Leipner, 2003). After fluorescence rise to a maximum intensity level (Fm), induced by high saturating light pulses (18,000 μE m^-2^ s^-1^, for 2.5 s), leaf is left for a slow fluorescence decay (Fd) to a steady state value (*F*s) in 3–5 min under continuous saturating light, reduced to 1200 μE m^-2^ s^-1^ to avoid photoinhibition (Rfd measurement). Due to preceding dark-adaptation fluorescence ratio Fv/Fm = (Fm- F_0_)/Fm corresponds to maximum potential photochemical efficiency of photosystem II. The fluorescence decrease ratio Rfd = (Fm-Fs)/Fs is the kinetics measured with continuous saturating light, therefore the stabilization time of *F*s value is dependent on an actual efficiency of electron transport and activation of all photochemical reactions in chloroplasts (Lichtenthaler et al., 2005).

The data was presented as the averaged values of 8 readings taken from the base part of two leaves from middle nodes (ear leaf and leaf above) from 4 plants for each symbiotic and drought or fertilization variant. Measurements of AM and NM plants were made in a staggered manner during full irradiance period of phytotron light regime.

### Light-Saturated Photosynthetic and Transpiration Capacity

Maximum leaf gas exchange capacity (light-saturated photosynthetic rate, Amax, μmol CO_2_ m^-2^ s^-1^ and maximum stomatal conductance to H_2_O, gs, mol H_2_O m^-2^ s^-1^) was determined with Q-Box CO650 Plant CO_2_ Analysis Package (Qubit Systems, CA), coupled with an infrared CO_2_ gas analyser. The stomatal aperture reached maximum after 20 min exposition to 3000 μmol m^-2^ s^-1^ photosynthetic photon-flux density.

At each sampling day, readings were taken from the same 4 plants for each symbiotic and drought or fertilization variant and covered the base area of two middle leaves (9 cm^2^ of each sampling area). Measurements of mycorrhizal and NM plants were made in a staggered manner during full irradiance period of phytotron light regime.

Photosynthetic nitrogen (PNUE) or phosphorus (PPUE) use efficiency is the ratio of light-saturated CO_2_ assimilation rate to leaf N or P-content (expressed as weight units per leaf dry mass, g 100g^-1^).

### Leaf and Root Water Potential and RWC

Midday leaf and root water potential was measured according to modified Porcel and Ruiz-Lozano (2004) method with a C52 thermocouple psychrometer (Wescor, Inc., United States). Three leaf disks about 5 mm in diameter taken from lower leaves and three 5 mm long fragments of secondary feeders roots were cut and stored as time samples in liquid nitrogen. After thawing for 5 min at 35°C samples were sealed in the C-52 chamber. After 45 min incubation, necessary for vapor pressure stabilization within the chamber, the readings were recorded by the Wescor PSYPRO^TM^ microvoltometer.

Relative water content (RWC) – normalized to DW, ratio of fresh weight (FW) to weight of tissue in full turgor (TFW, after 4 h of saturation at 4°C): RWC = [(FW-DW)/TFW-DW)] × 100.

### Chemical Analysis of Green Fodder and Estimation of Maize Silage Nutritive Value

Chemical analysis of representative samples of fresh maize (chopped, mixed and dried stalks, leaves and ears) were done at the time of harvest, in accordance with the applicable AOAC^2^ standards or the Polish Standard in Department of Animal Nutrition Laboratory (Poznań University of Life Sciences, Poland). The data represent averaged values, taken as mixed samples (*n* = 4), from four separate treated plants for each fertilization and symbiotic variant.

Dry matter was analyzed in binder dryer according to AOAC 934.01 method. Crude protein (CP) and nitrogen content was measured by Kjeldahl method (AOAC 976.06) in Kjelfoss Automatic 16210. Crude fiber^3^ (CF) was analyzed by Tecator Foss Fibertec System M. Ether extract (EE) was analyzed according to Soxhlet method (AOAC 2003.06) and Tecator Soxtec System HT 1043. Crude ash (Ash) was collected after a sample was burnt in Nobertherm oven (550°C) (AOAC 942.05). Nitrogen free extract was calculated on the basis of chemical composition according to equation NFE = (100 – CP – CF – EE – Ash). In addition, the nutritive value of maize silage was calculated on laboratory analysis of fresh maize using the PrevAlim 3.23 software (Educagri/INRA, Theix, France).

Analyses were conducted using the appropriate procedures (PROC MEANS, PROC UNIVARIATE, PROC GLM) of SAS software. Statistical analyses were performed using the general linear models procedure and Duncan’s multiple range test. Differences were reported as significant when *P* ≤ 0.05.

## Results

### Optimization of Pot Culture Methods for Long-Term Fungal Vitality and Equal Productivity of AM and NM Plants

#### Evaluation of Corn Symbiotic N and P Absorption Efficiency Under Four Fertilization Regimes

**FIGURE 1:**
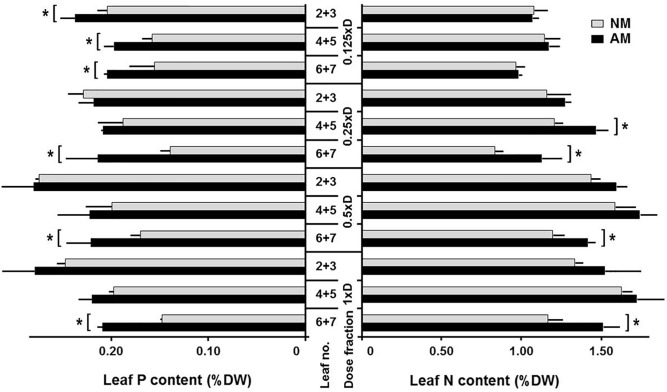
Effect of mycorrizae and fertilization level0 on nitrogen (N) and phosphorus (P) accumulation in leaves at three vertical canopy positions. P and N content of non-mycorrhized (NM) and mycorrhized (AM) plants, grown at four fertilization levels (0.125xD, 0.25xD, 0.5xD, 1xD). The data represent averaged values (*n* = 4), taken as mixed samples from two adjacent leaves (joined leaf numbers: L2+3, L4+5, and L6+7), representing four plants for each fertilization and symbiotic variant The measurements were made at the time of tasseling (10 weeks after sowing), after 4 weeks fertilization with one of four fertilizer dilution. The minerals content was expressed as weight units per leaf dry mass (g 100g^-1^). Asterisks represent statistical significance between NM and NM means according to *t*-Student test at *p* < 0.05.

To find the highest fertilization level being not detrimental for long-term hyphal vitality and maintaining at the same time similar shoot size and nutritional status of AM and NM counterparts, we evaluated mycorrhizal impact on leaf N and P accumulation (Figure 1) at the time of tasseling (BBCH stage 59, i.e., 10 weeks after sowing). After 6 weeks under low fertigation (0.25xD level, see below), required for advancement of fungal root colonization, the plants were provided for 4 weeks with one of four fertilizer dilutions (0.125xD, 0.25xD, 0.5xD, 1xD) for imitation of soil of fertility lowered by an equal decrease in each macro- and micronutrient. The 1xD fertilization level corresponds to 114:36:120:18 mg N:P_2_O_5_:K_2_O:MgO, as expressed in weekly doses per plant or 13.6 mM N and 0.85 mM Pi, according to two major components limiting fungal infection. Although the designed soil-free semi-hydroponic system and pots were irrigated with a water-soluble fertilizer, we expressed the nutrition rate also in relation to plant weight. In our opinion such a formulation not only allows greater precision for comparisons with pot experiments of other authors, but also enables approximation to field NPK weight fertilization units (see the fertilizer composition and discussion in Supplementary Data Sheet 1 “Hydroponics vs. field corn cultivation”).

In roots, there was no tendency for P and N accumulation caused by fertilizer increase. The variances due to symbiosis were also small, as the average P content (% DW) was 0.24 ± 0.01 for NM and 0.27 ± 0.01 for AM, while the average N content was 1.09 ± 0.06 for both NM and AM. Leaf N and P absorption was studied at three leaf positions: upper, middle and lower (Figure 1, L2 + 3, L4 + 5, and L6 + 7 indicate pairwise collected leaves, counted from the top). Leaf P and N content responded to fertilizer increase at each leaf position, but the point of saturation was reached at 0.5xD fertilization level, above which accumulation growth decelerated. However, only the top two nutrition variants did not show visual N and/or P deficiency symptoms.

The lowest fertilization regime (0.125xD) was the only one with AM-dependent P accumulation significantly higher at each leaf position (Figure 1). The difference in P responsivity between AM and NM decreased with increasing dose. As a result, under high fertilization only for the older leaves (L6+7) the mycorrhizal enhancement was observed, whereas for upper or middle leaves the difference between AM and NM plants was not statistically significant. Similar AM compensation for older leaves was related to N content. Mycorrhiza increased leaf N acquisition mainly at lower fertilization levels, but this effect became significant starting from the 0.25xD dose. It means that the threshold for N availability was at least several-fold higher than for P.

#### Fertilizer Effect on Hyphal, Arbuscular and Vesicular Abundance

The *Rhizophagus irregularis* vaccine was a suspension of fungal spores taken from monoxenic root cultures (TERI, New Delhi). The inoculum production involved optimization of spore germination under aseptic conditions, so it provided high intraradical hyphal growth and stable vitality. Figure 3A summarizes the parameters of fungal root colonization, examined against a wide range of fertilizer dilutions applied until 10th week of corn culture growth. The 1xD dilution (13.6 mM N and 0.86 mM Pi) was the highest fertilizer concentration found as not detrimental to long-term hyphal vitality and was eventually chosen for further drought experiments. However, during the first 6 weeks after sowing (until the 6-leaf stage), the fertilizer volume had been reduced to 0.25xD level in order to prevent inhibition of fungal root infection.

**FIGURE 2:**
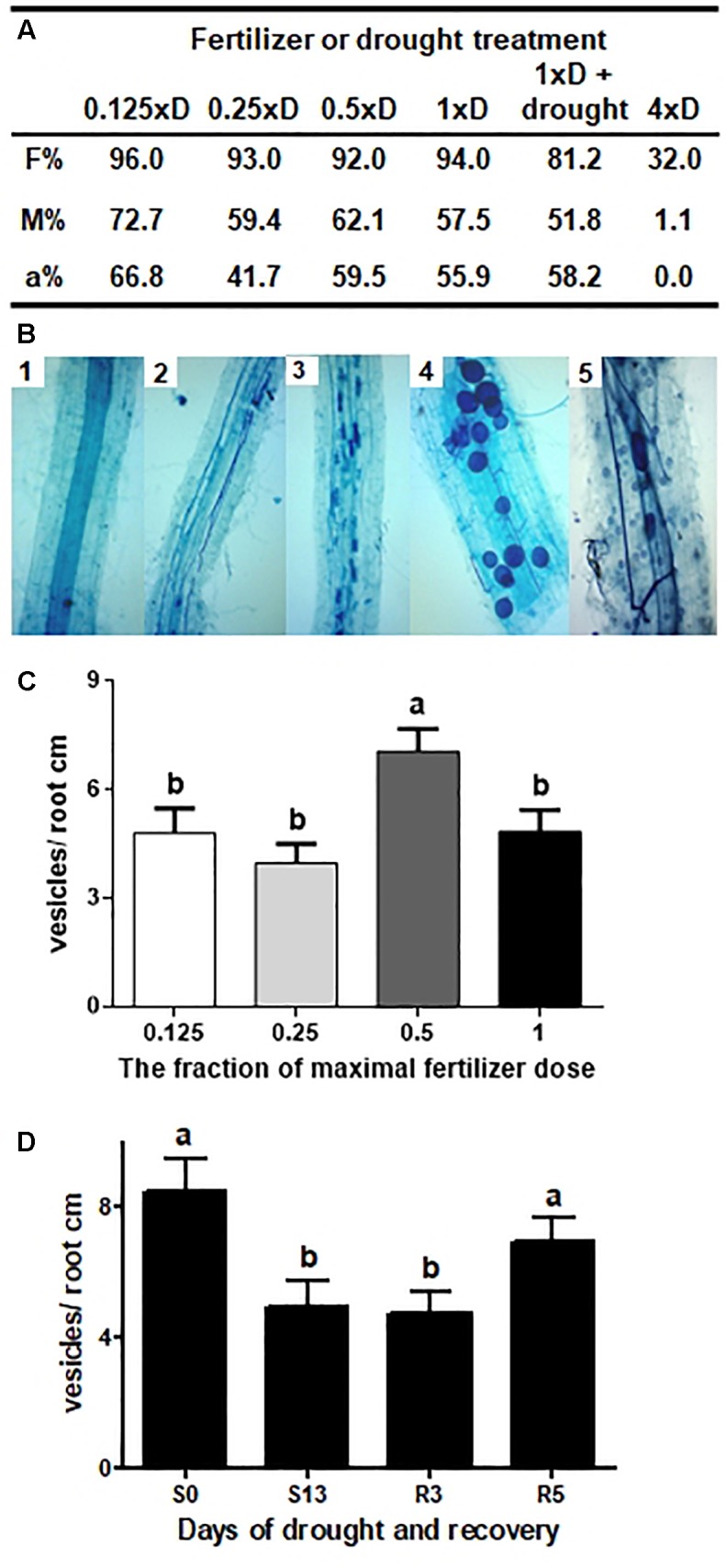
Long-term intraradical hyphae vitality under different fertilization and drought regimes. **(A)** Fertilizer or drought effect on hyphal root colonization and arbuscules abundance. F% – frequency of fungal structures in the root system, M% – intensity of the mycorrhizal colonization, a% – arbuscular abundance in colonized parts of root fragments, after 6 weeks on 0.25xD fertilizer dilution and additional 4 weeks on fertilization levels annotated in the table (10th week after sowing). Drought impact was examined after additional 2 weeks of drought and 1 week-long recovery; **(B)** Developmental stages and hyphae vitality. 1: non mycorrhized control roots, 2 and 3: acceleration of arbuscules development between 6th and 8th week of growth on 0.25xD fertilization level, 4: vesicles abundance after additional 4 weeks on 1xD dose, 5: sporulation level at the time of silage harvest (20th week after sowing); **(C)** Effect of fertilizer dose on AM vesicles abundance, after 6 weeks on 0.25xD fertilizer dilution and additional 4 weeks on fertilization levels annotated in the table (10th week after sowing); **(D)** Effect of drought and recovery on AM vesicles abundance, after 6 weeks on 0.25xD fertilizer dilution and additional 4 weeks on 1xD fertilization levels annotated in the table (10th week after sowing). Different letters symbolize statistically significant difference between means according to non-parametric Kruskal–Wallis one-way analysis of variance (*p* < 0.0001) followed by Dunn’s multiple comparisons test (*p* < 0.01). The error bars show the standard error of the mean values (*n* = 300–400).

Until 6th week, the progress of fungal root colonization did not exceed 30% (parameter F%), while arbuscular abundance (parameter a%), used as the hyphal vitality index, was negligible. Observing the effects of 1xD fertilizer dilution, we noticed that both indices reached their maxima during the following 2 weeks, when the planned increase in fertilization rate had already been executed (the acceleration of arbuscule development was illustrated in Figure 2C,D). Such high values of fungal parameters persisted until 10th week of cultivation (tasseling stage, BBCH 59), when the evaluation of fertilization limits was terminated. In comparison, the much more concentrated 4xD fertilizer solution (54.3 mM N, 3.4 mM Pi) was found to limit arbuscule development, although the hyphae still kept some potential (M% = 1.1) to infect at least 30% of roots (Figure 2A).

It can be therefore considered that nutrient application up to the 1xD level was not a factor affecting intraradical hyphal growth rate, formation of arbuscules and vesicles, and their long-term maintenance. This conclusion was further supported during later experiments, when moderately (0.5xD) or highly fertilized (1xD) AM corn cultures were grown for 20 weeks until the grain-filling stage (see root sample in Figure 2B). However, the reduction of P application down to 60% of recommended field levels seems to be a restriction necessary to maintain symbiosis in AM pot cultures (see calculations in Supplementary Data Sheet 1 “Hydroponics vs. field corn cultivation”).

No less importantly, this step was also an attempt to show a correlation between increased levels of fertilization and the size of fungal nutrient deposits (intraradical vesicle number, Figure 2A). Such a correlation was apparent for the plant host, resulting in the accumulation of mineral compounds and leaf nitrogen index (NBI). However, in the case of mycelium, we found that only one fertilizer level (0.5xD) caused an increase in vesicle abundance, compared to the average of all variants.

#### Validation of Non-invasive Methods for Evaluation of Mycorrhiza-Altered Leaf Physiology and Nutritional Status

Non-destructive techniques were necessary for time-course evaluation of leaf physiology, planned for drought experiments. Biochemical analysis of stem nitrogen distribution showed that the ear leaf and the leaf above the cob (L4 + 5) are the most appropriate for validation of fluorimetric estimation of mycorrhiza-altered corn N management. The middle leaves were selected because of the highest N accumulation at this leaf position under each fertilization regime and N amount increasing proportionally with increased NPK doses (Figure 1). Besides, such a narrowed sampling reduced the variability at distant leaf positions associated with N status diagnosis.

**FIGURE 3:**
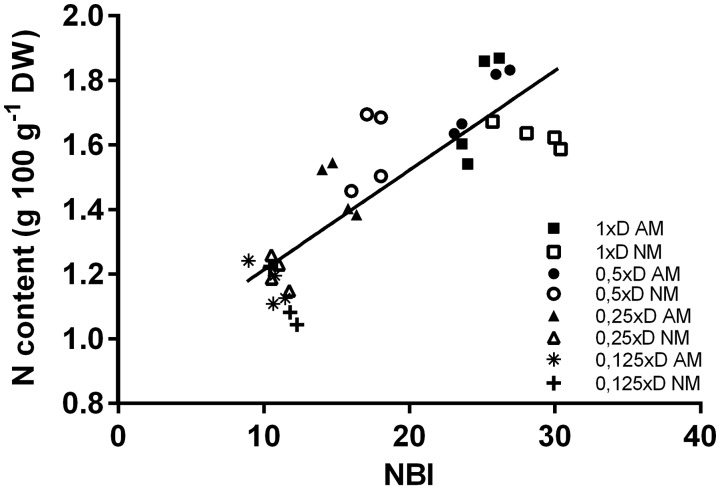
Correlation between the Nitrogen Balance Index (NBI) and the nitrogen content of the middle leaves. NM, non-mycorrhized plants; AM, mycorrhized plants. Data was collected for analysis (*n* = 32) from four fertilization variants (1xD, 0.5xD, 0.25xD, 0.125xD). Both nitrogen content (expressed as weight units per leaf dry mass) and NBI measurements were done on the same plants and leaves (L4 + 5, ear leaf and leaf above the ear). Pearson’s correlation coefficient (value *R*^2^) significant at the level of 0.01% (*p <* 0.0001).

**FIGURE 4:**
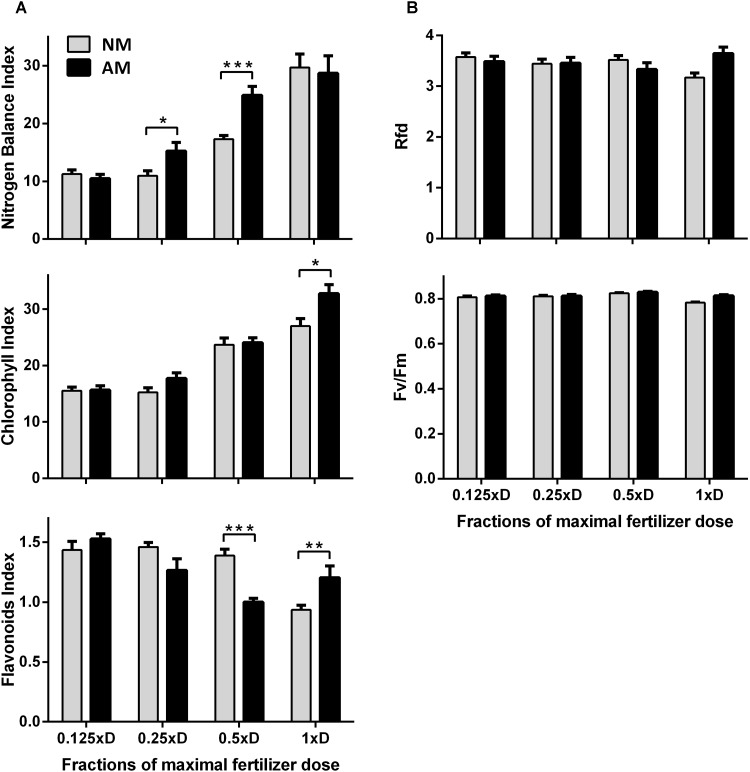
Effect of increasing fertilization on nitrogen status **(A)** and PSII quantum conversion **(B)** of the middle leaves, as indicators of nutritional status of NM and AM plants. Rfd, chlorophyll fluorescence decrease ratio; Fv/Fm, maximum quantum yield of PSII in the dark-adapted state. The bars represent average values taken from middle leaves (L4 + 5, ear leaf and leaf above) at the time of tasseling (10 weeks after sowing), after 4 weeks fertilization with one of 4 dilutions of maximal (1xD) dose. The error bars show the standard error of the mean (*n* = 80 for NBI, Chl and Flv: 4 plants × 2 leaves × 10 sampling points covering upper half of leaf length, *n* = 8 for Rfd, Fv/Fm: 4 plants × 2 leaves). Asterisks show statistically significant difference between NM and NM means according to *t*-Student test (*^∗^p <* 0.05, ^∗∗^*p* < 0.01, ^∗∗∗^*p* < 0.001).

A very useful tool for non-invasive estimation of leaf N management is the ratio of chlorophyll (Chl) to flavonoids (Flv) fluorimetric indices, called Nitrogen Balance Index (NBI). NBI was proportional (*R*^2^ = 0.72) to changes in total nitrogen level observed in leaves of plants growing at different dilutions of the medium (Figure 3). Similar to biochemical N determination, the NBI index reactivity was intensified by the presence of mycorrhiza only for moderate doses (0.25xD and 0.5xD), whereas the lowest dose contained apparently too little N to diversify these two symbiotic variants (Figure 4A). The high compatibility of the middle leaf NBI index with N content changes allowed us to accept its measurements as a reliable indicator of AM-affected N management efficiency. It was confirmed at the same time that the 1xD dose aligns the physiological performance of AM and NM plants in the early generative phase of growth.

Anticipating the requirements of drought experiments we tested the response of middle leaves also with respect to light-saturated photosynthetic efficiency parameters. The values of Rfd index (Figure 4B), corresponding to quantum conversion efficiency of photosystem II (see description in Section “Materials and Methods”), leaf CO_2_ exchange rate (Amax) and stomatal water conductance (gs) suggest that actual CO_2_ fixation capacity of AM and NM plants were comparable after long-term growth under medium (0.5xD) or high (1xD) fertilization (Table 1). Under these two fertilization levels not only net photosynthetic rate but also foliar N or P content were found to be similar in middle leaves (Figure 1), which led to no significant difference in PNUE or PPUE use efficiency with respect to AM symbiosis (Table 1).

**Table 1:** Effect of mycorrizae and fertilization level on light-saturated photosynthetic efficiency of the middle leaves.

	Fertilization	NM		AM	
Amax	0.5xD	16.9	±3.6	19.4	±2.9
	1xD	32.2	±7.2	30.5	±6.1
gs	0.5xD	0.11	±0.03	0.15	±0.03
	1xD	0.22	±0.06	0.29	±0.06
PNUE	0.5xD	10.6	±0.64	11.2	±0.46
	1xD	19.9	±0.69	17.7	±0.54
PPUE	0.5xD	76.1	±16	88.2	±13
	1xD	161	±36	154	±31


*Leaf CO_*2*_ exchange rate (Amax, μmol CO_*2*_ m^-*2*^ s^-*1*^) and stomatal water conductance (gs, mol H_*2*_O m^-*2*^ s^-*1*^), nitrogen (PNUE) or phosphorus (PPUE) use efficiency (the ratio of Amax to leaf N or P weight units) were measured on middle leaves (L4 + 5, ear leaf and leaf above) of AM and NM plants after long-term growth under medium (0.5xD) or high (1xD) fertilization. No statistically significant difference was found between the means of AM and NM.*

Unchanged Rfd indicated that the plants produced PSII centers, operating with full capacity, for each fertilizing level (Figure 4B). We show this parameter because its sensitivity has proved particularly useful for rapid and non-invasive detection of drought stress progress, when it comes to the reduction of the PSII quantum conversion capacity.

### Response of Fungal and Leaf Physiology to Drought Development and Recovery

**FIGURE 5:**
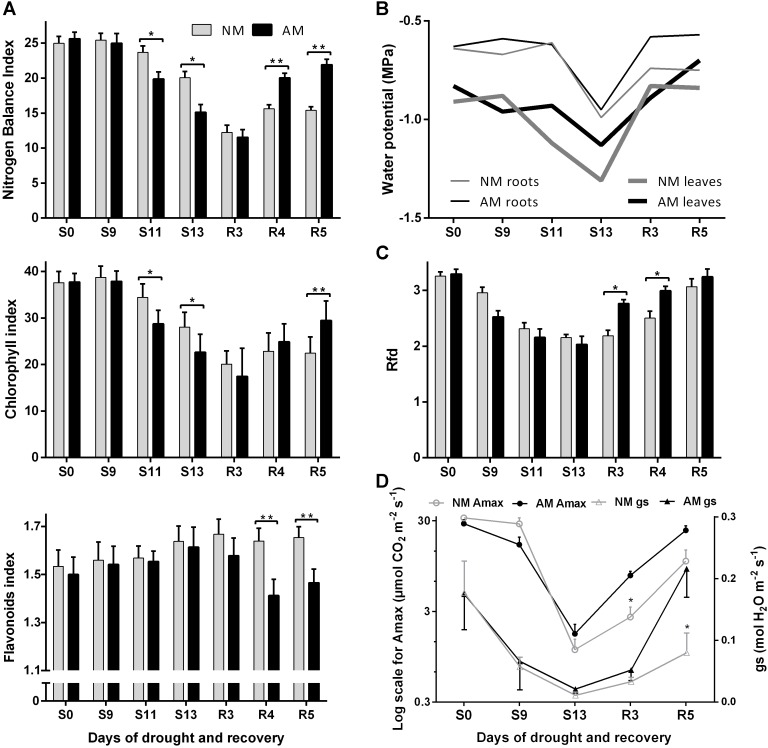
Effect of progressive drought and recovery on leaf nitrogen **(A)** and water **(B)** status, PSII quantum conversion **(C)** and light-saturated photosynthetic efficiency **(D)**. Rfd, chlorophyll fluorescence decrease ratio; Amax, light-saturated photosynthetic rate; gs, maximum stomatal conductance to H_2_O. Non-mycorrhized (NM) and mycorrhized (AM) plants were evaluated during full hydration (S0), drought conditions (S9, S11, S13) and rehydration (R3, R4, R5). The bars represent average values taken from combined measurements from the middle leaves (L4 + 5, ear leaf and leaf above); *n* = 100 for NBI, Chl and Flv: 5 plants × 2 leaves × 10 sampling points covering upper half of leaf length; *n* = 8 for Rfd, Amax, gs: 4 plants × 2 leaves. Asterisks show statistically significant difference between NM and NM means according to *t*-Student test (*^∗^p <* 0.05, *^∗∗^p <* 0.01, *^∗∗∗^p* < 0.001).

For purposes of plant drought response evaluation, a protocol mimicking field progressive drought stress and following recovery was established. We intended to assess cumulative severe stress effects, measured at the consecutive time points on the same leaf blades. Drought experiments were conducted on plants grown previously until silking (BBCH stage 63) under a fertilization regime (1xD) high enough to achieve similar shoot size and nutritional status of AM and NM plants (see details in Section “Results” subsections “Evaluation of Corn Symbiotic N and P Absorption Efficiency Under Four Fertilization Regimes” and “Fertilizer Effect on Hyphal, Arbuscular and Vesicular Abundance”). Growth conditions until this stage were also not limiting for hyphal vitality (Figure 2A,C). Soil drought was imposed by withholding watering for several days, followed by recovery (S0–S13 indicate days of drought, and R3–R5 are rehydration days, Figure 5B). Severe drop in root and leaf water potential (Ψ) was not registered until S13. The RWC (data not shown) in roots and leaves on that day (below 40 and 25%, respectively, data not shown) indicated that the Ψ decrease was caused mainly by dehydration. Both symbiotic variants, however, were equally able to restore Ψ and RWC values in both tissues within 2–3 days after rewatering.

Several non-destructive techniques were tested and adopted for purposes of leaf physiology evaluation under water stress. The most dynamic response was offered by fluorimetric indices: nitrogen management index (NBI) and chlorophyll fluorescence decrease ratio (Rfd) but also light-saturated photosynthetic rate (Amax) and light-saturated stomatal water conductance (gs). According to foregoing evaluation (see Section “Results” Subsections “Evaluation of Corn Symbiotic N and P Absorption Efficiency Under Four Fertilization Regimes” and “Validation of Non-invasive Methods for Evaluation of Mycorrhiza-Altered Leaf Physiology and Nutritional Status”), two adjacent middle leaves (L4 + 5, the ear leaf and the leaf above the cob) were chosen for time-course measurement of the aforementioned parameters. The response of leaf physiology and shift in leaf and root water potential allowed us to discriminate increasing, severe and decreasing phases of plant stress (Figure 5).

The symbiotic counterparts did not differ in decrease in photosynthetic conversion capacity (Rfd) and CO_2_ fixation (Amax) along with soil and leaf dehydration progress (Figure 5C,D, days S9–S13). The enhanced negative impact on nitrogen management efficiency (NBI) indicated AM-accelerated senescence rate during drought development (Figure 5A). Nevertheless, the Rfd index showed that AM plants rebuilt the photochemical potential within the first days of renewed watering (days R3 and R4), whereas the values for NM plants restored no sooner than after 5 days of rehydration (Figure 5C). The Chl index of both AM and NM plants continued to drop until 3rd day of rewatering (R3) which indicated delayed reversal of nitrogen-related senescence effects (Figure 5A). AM-dependent restoration of leaf nitrogen management appeared, however, more efficient, similarly to mycorrhizal impact on photosynthetic parameters. As a result, at 5th day of rehydration, the NBI value reached an almost 40% higher level in AM than in NM plants (Figure 5A). A more detailed analysis, considering senescence reversal rate, showed that at 7th day of rewatering, AM plants overwhelmed this ability of NM counterparts at each leaf position.

During drought development, the general response of stomatal gas exchange rate [CO_2_ assimilation (Amax) and water conductance (gs), both measured under saturated light] followed the pattern of fluorimetric and water potential variability in leaves (Figure 5D). After a large drop of those values in both symbiotic variants, the mycorrhizal influence was evident only during the rehydration period, when stomatal conductance and CO_2_ fixation capacity of AM plants restored faster.

### Evaluation of Long-Term Productivity of AM and NM Cultures Subjected to Transient Drought at the Grain-Filling Stage

To verify if mycorrhizae enhance corn fodder yield and/or nutritive value if highly fertilized pot cultures are subjected to drought, the plants were treated with NPK doses specific to field cultivation of silage corn (moderate 0.5xD or high 1xD) until the grain-filling stage (12 weeks after seeding). Drought was imposed for 2 weeks at the time of silking which is particularly sensitive, as it affects further grain development. Then fertilization was renewed for additional 5 weeks until the half-milkline stage of grain development recommended for silage harvest (BBCH stage 85). The plants were cultivated on the substrate taken from preceding AM or NM cultures (i.e., keeping the same composition, except the mycorrhizal component), diluted 1:15 (w/w) with sterile coconut-sand substrate.

Contrary to expectations, it turned out that mycorrhizal protection against drought stress was not sufficient to increase the final yield and food value (Supplementary Table 1). At harvest time, differences in growth parameters exceeded 10% (weight of leaves, shoots, and cobs), irrespective of symbiotic status, but for each dose the variants AM and NM did not differ significantly in growth parameters. The nitrogen index (NBI) for individual leaf positions on the shoot at harvest time was higher in more fertilized plants, on average by 40% in NM plants or 70% in AM plants (not shown). In contrast, differences in leaf NBI between symbiotic variants were not specific, and differed by up to 20% in favour of AM or NM plants in three independent cultures (not shown).

AM had no effect on organic nutrients of fresh corn as well as the estimated nutritive value of corn silage (Supplementary Table 1). Lower crude fiber content together with higher concentrations of crude protein and ether extract resulted in higher nutritive value, which increased with fertilization level. However, no statistically significant interaction between the effect of mycorrhiza and fertilization was observed.

## Discussion

### Fertilization Limits and Optima for Corn – *Rhizophagus irregularis* Symbiotic Coexistence and Final Productivity

Difficulty to achieve properly balanced fertilization, allowing to keep plants in good condition but low enough for initiation and conservation of hyphal infection, is a typical obstacle which should be overcome to establish mycorrhizal symbiosis both in pot cultures (Breuillin et al., 2010; IJdo et al., 2011; Balzergue et al., 2013; Bonneau et al., 2013) and in agricultural practice (Liu et al., 2000; Treseder and Allen, 2002; Oehl et al., 2003; Bhadalung et al., 2005; Schroeder and Janos, 2005; Vestberg et al., 2011; Verbruggen et al., 2013). One of the possible sources of discrepancy in nutrient (especially Pi) concentration, reported as critical for root AM colonization in soil-based pot cultures, is the risk of uncontrolled fertilizer concentration changes or even salts precipitation. Therefore for estimation of fertilization optima for long-term intraradical hyphal vitality we designed soil-free semi-hydroponic system (see details and comments in Supplementary Data Sheet 1 “Hydroponic vs. field corn cultivation”).

The negative effect of P fertilization on field AM hyphal growth and species diversity has been frequently reported (Liu et al., 2000; Treseder and Allen, 2002; Schroeder and Janos, 2005; Verbruggen et al., 2013), but AM fungal populations more adapted to high-input agroecosystems might be unaffected (Oehl et al., 2003; Bhadalung et al., 2005; Vestberg et al., 2011). For example, roots of resident plants in phosphate-polluted sites were found to be highly colonized, but plant performance is limited there due to relatively low soil N availability (Blanke et al., 2005, 2011). It is therefore not surprising that AM fungi are commonly present under moderate or high intensity of agricultural practices, including sites of continuous maize monocropping, even though species richness and abundance are severely affected (Oehl et al., 2003, 2005; Sheng et al., 2013). Nevertheless, it is still far from establishing more ‘mycocentric’ farming standards. Therefore, new crop management strategies need to be worked out to improve the composition of native or introduced AM fungal communities, especially considering the impact of fertilization, tillage and crop rotation on their symbiotic efficiency (Boomsma and Vyn, 2008; Gianinazzi et al., 2010; Smith and Smith, 2011; Verbruggen et al., 2013).

One of the most striking findings of this study was the preservation of high vitality of intraradical hyphae until corn maturity under fertilization regime approximating the nutrient recommendations for field cultivation of silage corn (see Figure 2A,C, and calculations in Supplementary Data Sheet 1 “Hydroponic vs. field corn cultivation”). Comparing our semi-hydroponic pot nutrition (expressed in weekly weight doses) with per plant NPK fertilization program for low-fertility soils, the only limitation was found in P application reduced to 60%. Furthermore, considering medium soil fertility prevailing in Poland, the Pi dosage needed to gain moderate corn yield seems to be very close to those we found as not detrimental for mycorrhizal mycelium development. This suggests that corn symbiosis with *R. irregularis* might be undisturbed under fertilization levels recommended in modern crop cultivation.

High NPK dosage (1xD fertilizer level) was also the one necessary to establish AM and NM cultures of similar size and nutritional status (Figure 1, 4A and Table 1). Growth on serial fertilizer dilution led to increased AM impact on leaf nitrogen and/or phosphorus accumulation (Figure 1), identifying the well-known mycorrhizal potential to compensate for low soil fertility. Under moderate or high fertilization rates, only the older leaves retained mycorrhizal enhancement at the time of tasseling, whereas for upper or middle leaves the difference between AM and NM plants was not significant statistically (Figure 1). The cultures supplied with 1xD fertilizer solution were set up for further evaluation of fungal drought potential in order to minimize interference from nutrient limitation.

Independently of the symbiotic status, the deposits of both mineral compounds showed diversity in relation to leaf position. Under each fertilization regime, the highest phosphorus level was found in top leaves, whereas the maximum nitrogen accumulation was noted in middle leaves (the ear leaf and the leaf above the cob). Such a targeted nutrient redistribution to the detriment of bottom leaves reflects optimization of photosynthetic efficiency of sun-exposed leaves and nutrient sink capacity of the ear leaf, necessary for grain filling. Shading by other leaves stimulates the process of nutrient recycling from bottom leaves, especially the remobilization of nitrogen, mainly from the photosynthetic apparatus (Miersch et al., 2000; Hörtensteiner, 2009).

Middle leaves (L4 + 5), showing the highest nitrogen accumulation and consistent response to soil fertility (Figure 1), were chosen for validation of a non-invasive method of leaf nitrogen status assessment based on chlorophyll (Chl) and flavonoids (Flv) fluorimetric indices (Figure 3, 4). The Chl:Flv ratio (NBI, Nitrogen Balance Index) is an efficient tool to assess crop plant response to soil fertility or drought development (Cerovic et al., 2002; Cartelat et al., 2005; Meyer et al., 2006; Tremblay et al., 2007; Cerovic et al., 2012). We tested this technique for purposes of further non-destructive evaluation of time-course response to drought stress, since nitrogen shortage is a major regulator of leaf senescence (Wingler et al., 2006; Gregersen et al., 2013; Balazadeh et al., 2014).

The method is based on the finding that leaf nitrogen deficiency is associated with a decrease in fluorescence related to chlorophyll-protein complexes – the main leaf nitrogen deposit (Kant et al., 2010). To obtain more adequate estimations, Chl readings are normalized against Flv UV absorption – another indicator highly correlated with leaf N management. The increase in Flv index results from a decreasing demand for carbon skeletons under low soil nutrient availability or other stresses (Cartelat et al., 2005; Meyer et al., 2006). The accumulation of carbon-based flavonoids is a phenomenon analogous to an increase in available sugar level under nitrogen deficiency (Wingler et al., 2006), both caused by a reduced use of carbon for synthesis of nitrogen compounds.

**FIGURE 6:**
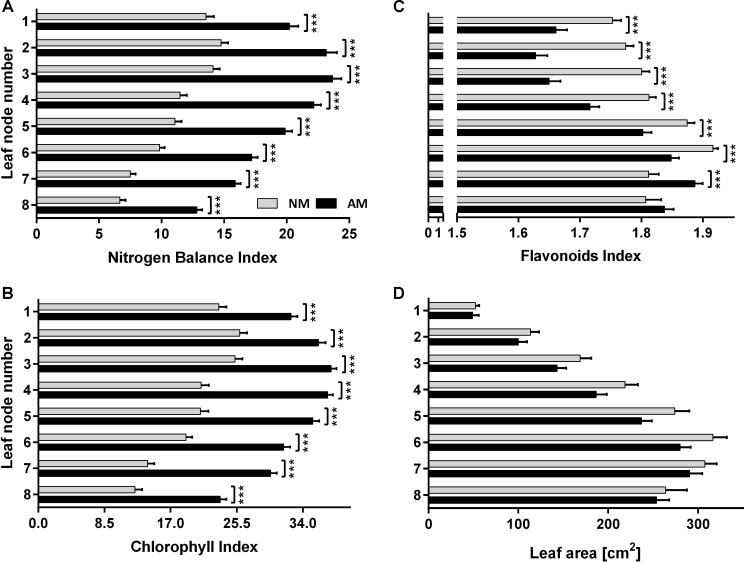
Leaf nitrogen status **(A)**, chlorophyll **(B)** and flavonoids **(C)** level and leaf area **(D)** at 8 leaf positions after 2-week drought imposed at silking time (10 weeks after sowing, fertilized with 1xD dose) and 7 days of rehydratation. The error bars show the standard error of the mean (*n* = 60 for NBI, Chl and Flv: 6 plants × 10 sampling points covering upper half of leaf length, *n* = 6 for leaf area measurements). Asterisks show statistically significant difference between NM and AM means according to *t*-Student test (*^∗^p <* 0.05, ^∗∗^*p* < 0.01, ^∗∗∗^*p* < 0.001).

The NBI index showed AM dependence at each leaf position (Figure 6, R7). In addition, in the ear leaf and the leaf above the cob (L4 + 5) the index was strongly correlated to applied fertilization levels (Figure 3, 4). Therefore NBI measurements at middle leaf positions can be recommended as a fast, non-invasive and reliable evaluation of mycorrhiza-altered corn nitrogen management. This finding is in accordance with Ciganda et al. (2009), who showed that non-invasive, reflectance-based measurement of Chl content in the collar leaf before silking or ear leaves explained more than 80 or 87%, respectively, of the variation in total Chl content in the corn canopy. The main component of the NBI index, which points to the better efficiency of N absorption in plants, turned out to be a lower Flv index (Figure 4A). This is reasonable under the assumption that the availability of soil nutrients was less reduced for AM plants, which in turn increased leaf carbon use (and lowered Flv readings) for synthesis of nitrogen compounds (Cartelat et al., 2005; Meyer et al., 2006).

In contrast to proportional plant response, the mycosymbiont was able to gather a clear surplus of hyphal vesicle number only under middle fertilization (Figure 2A,C). This indicated that the fungal pattern of nutrient accumulation was different from that of the plant host. According to the plant investment hypothesis, the long-term mycorrhizal dynamics is substantially dependent on photosynthetic carbon allocation and the plant host may adjust it when growth is N- or P-limited (Treseder and Allen, 2002; Treseder, 2004). It seems that proportionally growing plant nutritional benefit may be due to the fact that the plant becomes less and less dependent on the fungal acquisition system (Smith and Smith, 2012).

It is not surprising that crop varieties selected for high NPK assimilation rate are not simply AM-dependent, which means that in optimal soil conditions they do not require mycorrhiza to acquire a sufficient amount of mineral compounds. Modern crops, such as hybrid corn varieties, are rather expected to be AM-responsive than AM-dependent, exhibiting enhanced mycorrhizal growth mainly at low fertilizer levels, but with progressively reduced effect at high fertilization rates (van de Wiel et al., 2016; Lanfranco et al., 2018). This is understandable because with easier available resources the performance of the plant transport system may be as efficient as the fungal system (Smith and Smith, 2012).

### Indication of Mycorrhiza-Enhanced Reversal of Drought-Induced Leaf Senescence

Corn displays the isohydric strategy against drought, by reducing stomatal conductance and maintaining constant minimal daily leaf water potential and RWC (Tardieu, 1996). One of the constitutive traits improved in corn breeding programs is the so-called “stay green” behavior of delayed leaf senescence, which is also a feature of the hybrid evaluated in our study (‘Opoka’). This phenotype relies on stomata closure during drought and arresting of photosynthetic metabolism without losing chlorophyll (Hörtensteiner, 2009). The aim of our second experiment was to observe if progressive drought stress would alter leaf physiology of such a mutant.

Drought was imposed on AM and NM plants highly fertilized (1xD fertilizer level) until silking (BBCH stage 63) and hence of similar shoot size and nutritional status. In order to limit the effectiveness of stay-green corn phenotype we established an experimental set-up that allowed development of a severe but fully reversible drop in plant water potential. As a result of severe drought development even mycorrhization did not help the plant to avoid dehydration and sharp decline in leaf nitrogen status, PSII quantum conversion and light-saturated photosynthetic efficiency (Figure 5).

The decline in photosynthetic capacity followed by chlorophyll breakdown and degradation of pigment-binding proteins are common symptoms of genetically regulated progress of senescence (Miersch et al., 2000). In our previous study, both chlorophyll-related Nitrogen Balance Index (NBI) and the fluorescence decrease ratio (Rfd) were found to be sensitive markers of dark-induced senescence progression and reversal (Sobieszczuk-Nowicka et al., 2018). The steady-state measurement method, exposing the stressed leaves to saturating light, makes RFd index much better correlating with net CO_2_-fixation rates than the dark-adapted ratios (such as Fv/Fm) because it enables complete activation of photosynthetic processes (Lichtenthaler and Babani, 2004). Light-saturating conditions stimulate opening of stomata therefore Rfd value is a straight-forward indicator of the net CO_2_ assimilation rate and vitality index highly responsive to stress conditions (Lichtenthaler et al., 2005).

According to Rfd-values, the photosynthetic apparatus, independent of symbiotic status, becomes increasingly damaged under progression of drought, which was accompanied with a sharp drop in stomatal gas exchange parameters (Figure 5D). However, at the late phase of drought development, the leaf nitrogen management (NBI) of AM plants became negatively affected, as compared with their non-symbiotic counterparts (Figure 5A). It should be noted that fertilization had been suspended for the time of drought treatment. Therefore, it can be supposed that the available soil moisture and nutrients depleted faster from AM pots. The main factor responsible for this effect was AM-accelerated chlorophyll degradation progress (Chl index). In turn, enhanced accumulation of carbon-based flavonoids (Flv index) indicated that leaf soluble carbohydrates were in excess, compared to nitrogen availability in both symbiotic counterparts (Meyer et al., 2006; Cerovic et al., 2012).

AM plants often dry the soil at a faster rate than NM plants do (Ruiz-Lozano, 2003; Boomsma and Vyn, 2008; Bitterlich et al., 2018c). One of the reasons of this effect is the more active access of the root system to the soil solution, supported by hyphal penetration of small soil spaces. Altered soil water influx is also accompanied with higher stomatal conductance and transpiration in AM plants relative to NM plants, both under water-limiting and well-watered conditions (Augé, 2001; Augé et al., 2015). It is plausible that such enhancement of root and leaf water flux might exert a temporary negative effect on leaf physiology and explain our observation of AM-accelerated senescence rate during drought development. However, in field conditions such AM-dependent depletion of nutrients would not be expected since the fungal mycelium system could penetrate the soil unlimited by pot volume.

Nevertheless, mycorrhizal contribution became evident at the time of renewed watering, when faster restoration of leaf pigment, nitrogen and photosynthetic status was observed (Figure 5). During the rewatering period, the Chl:Flv ratio (NBI) reversed significantly faster in AM plants (Figure 5A). This increase could be ascribed to more efficient restoration of the balance between the use of carbon (lower Flv index) and nitrogen (higher Chl index) compounds. The major regulator of leaf senescence is the low availability of nitrogen to roots (Gregersen et al., 2013). The process is additionally regulated by an increase in the ratio of sugars to nitrogen compounds in N-depleted leaves (Wingler et al., 2006). Nevertheless, if nutritional deprivation does not go too far, the process of chlorophyll apoprotein degradation could be reversed upon water and nutrient resupply (Balazadeh et al., 2014).

Water stress and the resultant nutrient deficiency usually accelerate senescence of lower leaves in the corn canopy. Leaf senescence induced by nitrogen starvation alone or in conjunction with drought deprivation triggers complex regulatory networks, involving internal regulatory factors (Gregersen et al., 2013; Jibran et al., 2013). This results in controlled remobilization of degraded chloroplasts and other leaf components, converting senescing leaves into a nutrient source for younger leaves and generative organs as dominant sink tissues (Miersch et al., 2000; Hörtensteiner, 2009). This effect was also evident in our study but independent of the plant symbiotic status. Nevertheless, we found that *R. irregularis* enables corn plants a more extensive senescence reversal at each leaf position. As a result, at 7th day of the rehydration period, NBI reached an approximately two-fold higher level for AM than for NM plants (Figure 6A). The effect of mycorrhizae on restoration of leaf carbon use (Flv index) was not significant only for bottom leaves (Figure 6C).

One of the plausible explanations of AM-accelerated stress recovery is an immediate action of the fungal transport system, once watering was turned back, whereas water soil resources were not yet available to NM roots (Bitterlich et al., 2018a,c). An alternative explanation would be a better condition of AM than NM plants, as AM enables more active water acquisition after drought cessation, although not necessarily involving the fungal pathway (Augé, 2001). Similar AM and NM shoot size and leaf nutritional status under normal watering, designed in our model, seems to minimize the second option. Therefore we suggest that mycorrhizal effect was not mediated by nutritional supplementation before the stress but was triggered by mycelial transport activated at the time of recovery. From drought-unaffected arbuscule frequency it can be deduced that intraradical mycelium has retained this pathway until the time of rewatering. As a result, AM roots might be able to overtake the effectiveness of water and mineral compound flow in NM counterparts whose root tissues are the sole accessible transport pathway. Interestingly, the fungal potential was shown to be preserved at the expense of lowering AM vesicle number (Figure 2D). It can be interpreted as engagement of hyphal nutritional resources targeted to maintain the symbiotic relationship despite the reduced vitality of the host.

The more rapid recovery of NBI but also stomatal water conductance (gs) in AM plants were accompanied with a significant enhancement of light-saturated photosynthetic capacity parameters Rfd and Amax (Figure 5). In conclusion, it seems that enhanced reversal of stress-induced senescence and of stomatal resistance at the time of rewatering was the most prominent manifestation of mycorrhizal potential against detrimental drought effects. It could be recognized as the hallmark of better drought tolerance, which is defined as survival or specific behavior at low internal water potential or even dehydration (Augé, 2001).

Augé et al. (2015) meta-analysis shows that AM alteration of stomatal conductance can occur even if AM and NM plants do not differ in size or tissue P concentration. Therefore the notion that AM effects on plant water relations were mainly nutritional in nature, prevalent in the early literature, cannot be treated as a general rule. Nevertheless, the cited authors conclude that researchers can expect to see more mycorrhizal effects on leaf water management when experiments are conducted in nutritionally deficient conditions. Our results show that even if corn cultures are grown under non-limiting fertilization, the drought-induced senescence and stomatal closure can be altered by hyphal activity in a way apparently not mediated by AM-improved growth.

### Drought Alleviation by AM Did Not Affect the Final Productivity of Highly Fertilized Corn Cultures

The knowledge of AM effects on growth to normal reproductive stages and harvest times is comparatively limited (Smith and Smith, 2011) which also the case of mycorrhized maize investigation (Boomsma and Vyn, 2008). Therefore we evaluated the productivity of AM and NM counterparts fertilized with moderate or high NPK doses until the grain-filling stage. To imitate field drought constraints, the cultures were subjected to 2-week progressive drought at the time of silking. The quality and quantity of grains in the cob is the factor primarily affecting the nutritional value of corn crop harvested as fodder. For this reason, the drought was imposed when the corn was particularly sensitive to water scarcity (when pollination begins and ear silks begin to emerge, BBCH stage 63), which disrupts grain development (Subramanian et al., 1995; Boomsma and Vyn, 2008).

Contrary to expectations, the final yield and nutritional value of green forage in relation to NM plants were negligible (Supplementary Table 1). This means that alleviation of drought-induced senescence by AM was not sufficient to have a significant positive effect on long-term productivity of NM plants. This finding could be explained in various ways. Firstly, it might result from non-optimal genotype combinations of the plant and fungal strain or isolate (Lanfranco et al., 2018). Secondly, the presence of the fungal partner might suppress the plant P uptake pathway, whereas the mycorrhizal pathway functions not better than in non-symbiotic counterparts (Smith and Smith, 2011, 2015). Thirdly, the lack of measurable profits for the plant host might be the net effect of nutritional costs and benefits of in fact bilateral but hidden exchange (Smith and Smith, 2012, 2015). Fourthly, for corn stay-green hybrids the critical seasonal nutritional demands occur during the grain-filling stage, but nitrogen in this period is acquired mainly from soil resources rather than from plant compounds accumulated in the vegetative biomass before the flowering phase (Szulc et al., 2012). Finally, more advantageous mycorrhizal effects on crop quantity and quality can be expected either under lower soil nutritional value or when plants are exposed to a much longer period of water shortage.

All or some of these circumstances might be abolished by mycorrhiza-related growth response in terms of plant biomass or mineral nutrient transfer. It should be noted, however, that neutral mycorrhizal outcome does not unequivocally indicate a truly commensal or parasitic relationship (Smith and Smith, 2011). Even if mycorrhiza does not affect overall growth or mineral uptake by the plant, the mycelium – if present in the root cortex – still has a potential to provide an alternative route for water and nutrition supply. Such a hidden but mutualistic relationship may show up under abiotic stress situation, such as spring cold stress, salinity or soil water shortage, as it was demonstrated in this paper.

## Conclusion

Improved plant nutrition is the often suggested explanation of mycorrhizal effects on plant productivity and resulting enhancement of drought tolerance. This study shows that long-term corn symbiosis with *Rhizophagus irregularis* might be undisturbed under fertilization levels recommended for field cultivation of silage corn, but without any significant fungal effect on fodder yield and quality. Nevertheless, the reduced disproportion of nutritional status of AM and NM counterparts did not eliminate symbiotic benefits under challenging drought conditions. It was particularly remarkable at the time of rewatering, when mycorrhiza enhanced plant ability to reverse leaf senescence symptoms.

These findings are particularly interesting because the corn variety chosen for our study (‘Opoka’) is a stay-green hybrid. Such a phenotype is characterized by a lowered rate of developmental senescence during grain filling but additionally associated with enhanced drought resistance. Nevertheless, severe drought development in our study limited the effectiveness of this mechanism, and both AM and NM plants were not able to avoid dehydration. We can therefore conclude that genetically based timing and progression of drought-induced senescence could undergo alteration by hyphal activity even in stay-green mutants, moreover, in a way apparently not mediated by AM-improved growth.

## Author Contributions

EP-L conducted the experiments, analyzed the data, made illustrations, and contributed to writing. TL helped design conditions for pot cultures and analyzed the data. RM supervised fodder quality analyses. WP designed a research plan, conducted the experiments, supervised the work, and wrote the manuscript.

## Conflict of Interest Statement

The authors declare that the research was conducted in the absence of any commercial or financial relationships that could be construed as a potential conflict of interest.

## Footnotes

1 http://www2.dijon.inra.fr/mychintec/Mycocalc-prg/download.html

2 AOAC International, 2005. Official Methods of Analysis of the AOAC. 18th Edition, Gaithersburg, MD (United States)

3 PN-EN ISO 6865. Methods for analysis of animal feeding stuffs – Determination of crude fiber content – method with intermediate filtration
