# Supplementary material for: Arbuscular Mycorrhiza in Highly Fertilized Maize Cultures Alleviates Short-Term Drought Effects but Does Not Improve Fodder Yield and Quality
Source: Front Plant Sci. 2019 Apr 17;10:496. doi: 10.3389/fpls.2019.00496 (PMC6478757; doi:10.3389/fpls.2019.00496)
Supplement: Supplementary file 1 [file Table_1.docx]

Supplementary Table 1. Effect of mycorrhiza and fertilization level on green fodder chemical composition and nutritive value of maize silage.

|  |  |  | Chemical composition of maize green fodder* | | | | | | Maize silage nutritive value** | | | | |
| --- | --- | --- | --- | --- | --- | --- | --- | --- | --- | --- | --- | --- | --- |
| Group | Mycorrhiza | Fertilization level | DM  (%) | Ash  (%) | CP  (%) | CF  (%) | EE  (%) | NFE  (%) | UFL | UFV | PDIN  (g) | PDIE  (g) | PDIA  (g) |
| 1 | - | 0,5 | 28,1^a^ | 3,72^b^ | 4,51^c^ | 23,0^a^ | 1,83^c^ | 66,9 | 0,90^a^ | 0,79^a^ | 28,8^c^ | 59,3^d^ | 10,3^c^ |
| 2 | + | 0,5 | 26,8^a^ | 4,02^ab^ | 4,93^c^ | 22,1^a^ | 2,15^bc^ | 66,8 | 0,91^a^ | 0,80^a^ | 31,5^c^ | 60,5^cd^ | 11,3^c^ |
| 3 | - | 1 | 32,5^b^ | 3,73^b^ | 6,13^b^ | 20,9^ab^ | 2,49^ab^ | 66,7 | 0,92^a^ | 0,82^a^ | 39,2^b^ | 63,3^bc^ | 14,0^b^ |
| 4 | + | 1 | 27,9^a^ | 4,06^a^ | 6,74^b^ | 21,0^ab^ | 2,74^a^ | 65,4 | 0,92^a^ | 0,81^a^ | 43,3^b^ | 64,5^b^ | 15,0^b^ |
| 5 | - | 2 | 41,5^c^ | 2,82^c^ | 9,28^a^ | 17,3^c^ | 2,87^a^ | 67,8 | 0,97^b^ | 0,88^b^ | 59,0^a^ | 75,8^a^ | 21,3^a^ |
| 6 | + | 2 | 36,5^d^ | 3,05^c^ | 8,79^a^ | 19,4^b^ | 2,02^bc^ | 66,7 | 0,95^c^ | 0,85^c^ | 56,3^a^ | 74,3^a^ | 20,0^a^ |
| SEM |  |  | 1,15 | 0,104 | 0,386 | 0,455 | 0,100 | 0,314 | 0,006 | 0,007 | 2,46 | 1,39 | 0,888 |
| *P-value* |  |  | <0,01 | <0,01 | <0,01 | <0,01 | <0,01 | 0,489 | <0,01 | <0,01 | <0,01 | <0,01 | <0,01 |
| Main effects | - |  | 34,0a | 3,42a | 6,64 | 20,4 | 2,40 | 67,1 | 0,93 | 0,83 | 42,3 | 66,1 | 15,2 |
|  | + |  | 30,4b | 3,71b | 6,82 | 20,8 | 2,30 | 66,3 | 0,92 | 0,82 | 43,7 | 66,4 | 15,4 |
|  |  | 0,5 | 27,5^a^ | 3,87^a^ | 4,72^a^ | 22,5^a^ | 1,99^a^ | 66,9 | 0,90^a^ | 0,80^a^ | 30,1^a^ | 59,9^a^ | 10,8^a^ |
|  |  | 1 | 30,2^b^ | 3,89^a^ | 6,43^b^ | 21,0^b^ | 2,61^b^ | 66,1 | 0,92^a^ | 0,81^a^ | 41,3^b^ | 63,9^b^ | 14,5^b^ |
|  |  | 2 | 39,0^c^ | 2,94^b^ | 9,04^c^ | 18,3^c^ | 2,45^b^ | 67,2 | 0,96^b^ | 0,86^b^ | 57,6^c^ | 75,0^c^ | 20,6^c^ |
| Mycorrhiza |  |  | <0,01 | <0,01 | NS | NS | NS | NS | NS | NS | NS | NS | NS |
| Fertilization level | |  | <0,01 | <0,01 | <0,01 | <0,01 | <0,01 | NS | <0,01 | <0,01 | <0,01 | <0,01 | <0,01 |
| Mycorrhiza*Fertilization level | |  | NS | NS | NS | 0,09 | <0,01 | NS | NS | 0,09 | NS | NS | NS |

* Chemical composition parameters: DM – dry matter, CP – crude protein, CF – crude fiber, EE – ether extract, NFE – nitrogen free extract

** Nutritive value parameters: UFL – feed unit for lactation (1700 kcal net energy for lactation), UFV – feed unit for maintenance and meat production (1820 kcal net energy for fattening), PDIN – dietary protein undegraded in the rumen, but truly digestible in the small intestine and microbial protein which could be synthesized in the rumen from degraded dietary nitrogen, when energy and other nutrients are not limiting; PDIE – dietary protein undegraded in the rumen, but truly digestible in the small intestine and microbial protein which could be synthesized in the rumen from the energy available in the rumen, when degraded nitrogen and other nutrients are not limiting, PDIA - protein digested in the small intestine supplied by rumen-undegraded dietary protein

The nutritive values were calculated on the basis of the analyzed nutrient contents using the PrevAlim 3.23 software (Educagri/INRA, Theix, France)

^a,b^ Means within a column with different superscripts differ (P ≤ 0.05) NS – without differences (P> 0.05) SEM - standard error of the mean
