# Supplementary material for: Arbuscular Mycorrhiza in Highly Fertilized Maize Cultures Alleviates Short-Term Drought Effects but Does Not Improve Fodder Yield and Quality
Source: Front Plant Sci. 2019 Apr 17;10:496. doi: 10.3389/fpls.2019.00496 (PMC6478757; doi:10.3389/fpls.2019.00496)
Supplement: Supplementary file 2 [file Data_Sheet_1.docx]

**Hydroponic AM-optimal nutrition as compared to fertilization recommended for field corn cultivation**

The aim of experiments described in section 1a and 1b of Results was to define fertilization limits and optima for long-term intraradical hyphal vitality and symbiotic corn coexistence until the early generative stage. This objective has been achieved by the relatively high fertilizer application rate (1xD, see below for nutrient composition details). Although up to 6^th^ week the nutrition was low (0.25xD), the later fertilization shift constantly exposed the symbiotic stability to the possibility of weakening. Nevertheless, we found that vitality of the mycelium was not diminished even in case of prolonged cultivation necessary to evaluate the influence of AM on green fodder yield. In this case, the pot cultures required on average 20 weeks to achieve the adequate grain maturity (see further details in Results section 3). This was particularly puzzling in the context of the lack of restrictions on mycelium vitality, which we expected with such a high concentration of mineral ingredients in the fertilizer solution. According to two major components limiting fungal infection it was equal to 13.6 mM N and 0.85 mM Pi. This is equivalent to 0.9x N and 0.85x P fraction of Hoagland’s formula (Hoagland and Arnon, 1950) and it goes far beyond the 0.1-0.5 mM Pi concentration range often suggested as critical for maintaining efficient mycorrhizal symbiosis in pot cultures (Balzergue et al., 2013, Bonneau et al., 2013, Breuillin et al., 2010, IJdo et al., 2011). See Table 1 for several examples of P limitation reported for mycorrhizal pot cultures of crop plants from recent studies and Discussion section for comments of other authors works addressing fertilization limits for field-grown AM plants.

The risk of uncontrolled fertilizer concentration changes or even salts precipitation in pot substrate is common to soil-based cultures and might be the cause of discrepancies in fertilizing limits reported for mycorrhizal symbioses. Therefore we designed a soil-free semi-hydroponic system with a mixture of coconut fiber and sand, and frequent irrigation with a water-soluble fertilizer. Most of laboratory growth experiments report fertilization of mycorrhizal cultures with nutrient solution concentration units. However, such a formulation is adequately precise only for fully hydroponic systems, when the fertilizer solution is frequently changed. In contrast, fertilizer concentration formula in soil-based substrates might be misleading, as it is in case of inaccurate estimation of the P-fixing capacity of the pot substrate devoid of hyphal activity. Moreover, caution is required when interpreting mycorrhizal experiments with restricted fertilization or limited pot volume. Such factors might be sources of discrepancy in Pi concentration reported as critical for root colonization and long-term hyphal vitality.

Our observations led us to compare NPK doses applied in our pot cultures to those recommended for field corn cultivation. In the experiments we used an easily soluble commercial fertilizer (Kristalon Blue, Yara Poland) with a lowered P content (NPKMg 19:6:20:3). Kristalon Blue is produced with the same mineral components as YaraMila fertilizers recommended by Yara Poland for field corn cultivation. The coconut fiber/sand substrate corresponds to characteristics of soils of very low fertility, for which the highest NPK fertilization should be applied. In such a case the manufacturer recommends soil supplementation with 500 kg ha^-1^ of YaraMila CORN (NPKMg 7:20:28:2) applied before sowing and 400 kg ha^-1^ of YaraMila SuperCAN (NPKMg 27:5:5:0) applied in the split dose system^[[1]](#footnote-1)^. According to this nutrition program, the fertilization level should reach 140:120:160:11 kg N:P_2_O_5_:K_2_O:MgO ha^-1^, but only a moderate corn yield could be expected on poor soils.

The corn hybrid (‘Opoka’) used in our study was bred specifically for silage production, and the breeder (HR Smolice, Poland)^[[2]](#footnote-2)^ recommends planting densities of 10-11/m^2^. Taking into account such a density and assuming that field cultivation would last as long as pot cultures (20 weeks), we can convert field fertilization values into per plant weight dose that we applied weekly in pot experiments. Such roughly estimated field units correspond to 72:60:80:6 mg N: P_2_O_5_:K_2_O:MgO, as expressed in weekly doses per plant. In the pot cultures, the highest weekly fertilizer dose per plant (1xD) being not detrimental for long-term hyphal vitality was 114:36:120:18 mg N:P_2_O_5_:K_2_O:MgO. Comparing both fertilization regimes it seems that the only restriction necessary to maintain symbiosis in AM pot cultures is a reduction of phosphorus application down to at least 60% of the level recommended for low-fertility soils. Moreover, according to foregoing calculations, at soils of medium fertility, prevailing in Poland, a moderate corn yield might be achieved^[[3]](#footnote-3)^ with application of 70-80 kg P_2_O_5_ ha^-1^, which is very close to the P_i_ dose non-inhibiting for mycorrhizal mycelium development in our pot experiments.

Obviously, the foregoing recalculation is only approximate, since in pot cultures the fertilizer was delivered in equal weekly doses whereas under field conditions macronutrient availability undergoes seasonal fluctuations. On the one hand, foliar N or P fertilizer application, recommended for weak soil types or to reduce spring cold stress, might be temporarily unfavorable for stability of mycorrhizal association. On the other hand, losses of available soil nutrient residues should be expected during the growing season due to nitrate leaching and poor phosphorus mobility. Nutrient loss from the system might be, however, alleviated by a highly efficient mycorrhizal pathway, which greatly increases the absorbing root surface area. Extraradical hyphal network extending beyond the root depletion zone almost entirely contributes to Pi acquisition of AM plants but also provides access to soil nitrogen acquired from both inorganic and organic forms (Hodge and Storer, 2015, Lanfranco et al., 2018, Miller, 2000).

Table 1. Maximal N and P fertilization levels reported in recent papers as not detrimental for AM root colonization rate

|  | **0.125 xD** | **0.25 xD** | **0.5 xD** | **1xD** | **Hoagland** | **Auge et al. 2008** | **Moche et al. 2010** | **Barzana et al. 2012** | **Bonneau et al. 2013** | **Breuillin et al. 2010** |
| --- | --- | --- | --- | --- | --- | --- | --- | --- | --- | --- |
| **N-NO3** | 1.06 | 2.13 | 4.25 | 8.5 | 14 | 7.5 | 5.0 | 15.0 | 1.2 | 5.0 |
| **N-NH4** | 0.63 | 1.27 | 2.53 | 5.07 | 1.0 |  |  |  |  | - |
| **P-PO4** | 0.11 | 0.21 | 0.43 | 0.85 | 1.0 | 0.6 | 0.1 | 0.25 | 0.13 | 0.2 |

Data expressed in concentration (mM) units to allow comparison to Hoagland formula used for hydroponic cultures.

**References**

Augé, R. M., Toler, H. D., Sams, C. E., Nasim, G. (2008). Hydraulic conductance and water potential gradients in squash leaves showing mycorrhiza-induced increases in stomatal conductance. Mycorrhiza. 18, 115-121. doi: 10.1007/s00572-008-0162-9

Balzergue, C., Chabaud, M., Barker, D. G., Bécard, G., Rochange, S. F. (2013). High phosphate reduces host ability to develop arbuscular mycorrhizal symbiosis without affecting root calcium spiking responses to the fungus. *Front. Plant Sci.* 4**,** 426. doi: 10.3389/fpls.2013.00426

Bárzana, G., Aroca, R., Paz, J. A., Chaumont, F., Martinez-Ballesta, M. C., Carvajal, M. Ruiz-Lozano, J. M. (2012). Arbuscular mycorrhizal symbiosis increases relative apoplastic water flow in roots of the host plant under both well-watered and drought stress conditions. Ann Bot, 109, 1009-1017. doi: 10.1093/aob/mcs007

Bonneau, L., Huguet, S., Wipf, D., Pauly, N., and Truong, H.-N. (2013). Combined phosphate and nitrogen limitation generates a nutrient stress transcriptome favorable for arbuscular mycorrhizal symbiosis in Medicago truncatula. New Phytol. 199, 188–202. doi: 10.1111/nph.12234

Breuillin, F., Schramm, J., Hajirezaei, M., Ahkami, A., Favre, P., Druege, U., Hause, B., Bucher, M., Kretzschmar, T., Bossolini, E. (2010). Phosphate systemically inhibits development of arbuscular mycorrhiza in Petunia hybrida and represses genes involved in mycorrhizal functioning. *Plant J*. 64**,** 1002-1017. doi: 10.1111/j.1365-313X.2010.04385.x

Hoagland, D. R. and Arnon, D. I. (1950). The water-culture method for growing plants without soil. *Circ. – Calif. Agric. Exp. Stn*. 347.

Hodge, A. and Storer, K. (2015). Arbuscular mycorrhiza and nitrogen: implications for individual plants through to ecosystems. *Plant Soil*. 386**,** 1-19. doi: 10.1007/s11104-014-2162-1

IJdo, M., Cranenbrouck, S., Declerck, S. (2011). Methods for large-scale production of AM fungi: past, present, and future. *Mycorrhiza*. 21**,** 1-16. doi: 10.1007/s00572-010-0337-z

Lanfranco, L., Fiorilli, V., Gutjahr, C. (2018). Partner communication and role of nutrients in the arbuscular mycorrhizal symbiosis. *New Phytol*., 220, 1031-1046. doi: 10.1111/nph.15230

Miller, M. H. (2000). Arbuscular mycorrhizae and the phosphorus nutrition of maize: a review of Guelph studies. *Can. J. Plant Sci*. 80**,** 47-52. doi: 10.4141/P98-130

Moche, M., Stremlau, S., Hecht, L., Göbel, C., Feussner, I., Stöhr, C. (2010). Effect of nitrate supply and mycorrhizal inoculation on characteristics of tobacco root plasma membrane vesicles. Planta, 231, 425. doi: 10.1007/s00425-009-1057-5

1. <https://www.yara.pl/odzywianie-roslin/kukurydza/maize-fertilizer-programs/> [↑](#footnote-ref-1)
2. <http://www.hrsmolice.pl/images/kukurydza/karty/opoka.pdf> [↑](#footnote-ref-2)
3. <https://www.ior.poznan.pl/plik,1428,integrowana-produkcja-kukurydzy-pdf.pdf> <https://www.yara.co.uk/crop-nutrition/forage-maize/increasing-forage-maize-yield/> [↑](#footnote-ref-3)
